# Blood groups of Neandertals and Denisova decrypted

**DOI:** 10.1371/journal.pone.0254175

**Published:** 2021-07-28

**Authors:** Silvana Condemi, Stéphane Mazières, Pierre Faux, Caroline Costedoat, Andres Ruiz-Linares, Pascal Bailly, Jacques Chiaroni

**Affiliations:** 1 Aix Marseille Univ, CNRS, EFS, ADES, UMR 7268, Marseille, France; 2 Department of Genetics, Evolution and Environment, and UCL Genetics Institute, University College London, London, United Kingdom; 3 Ministry of Education Key Laboratory of Contemporary Anthropology and Collaborative Innovation Center of Genetics and Development, School of Life Sciences and Human Phenome Institute, Fudan University, Yangpu District, Shanghai, China; 4 Établissement Français du Sang PACA-Corse, « Biologie des Groupes Sanguins », Marseille, France; University of Florence, ITALY

## Abstract

Blood group systems were the first phenotypic markers used in anthropology to decipher the origin of populations, their migratory movements, and their admixture. The recent emergence of new technologies based on the decoding of nucleic acids from an individual’s entire genome has relegated them to their primary application, blood transfusion. Thus, despite the finer mapping of the modern human genome in relation to Neanderthal and Denisova populations, little is known about red cell blood groups in these archaic populations. Here we analyze the available high-quality sequences of three Neanderthals and one Denisovan individuals for 7 blood group systems that are used today in transfusion (ABO including H/Se, Rh (Rhesus), Kell, Duffy, Kidd, MNS, Diego). We show that Neanderthal and Denisova were polymorphic for ABO and shared blood group alleles recurrent in modern Sub-Saharan populations. Furthermore, we found ABO-related alleles currently preventing from viral gut infection and Neanderthal *RHD* and *RHCE* alleles nowadays associated with a high risk of hemolytic disease of the fetus and newborn. Such a common blood group pattern across time and space is coherent with a Neanderthal population of low genetic diversity exposed to low reproductive success and with their inevitable demise. Lastly, we connect a Neanderthal *RHD* allele to two present-day Aboriginal Australian and Papuan, suggesting that a segment of archaic genome was introgressed in this gene in non-Eurasian populations. While contributing to both the origin and late evolutionary history of Neanderthal and Denisova, our results further illustrate that blood group systems are a relevant piece of the puzzle helping to decipher it.

## Introduction

Over the last decade, technological progress has allowed generation of data from the entire genome of some fifteen extinct Neanderthal and Denisova hominins who lived 40,000 to 100,000 years ago from Western Europe to Siberia [1]. It reveals population structure, several demographic fluctuations and gene flows across hominin populations, worldwide dispersal of archaic genes by admixed *Homo sapiens*, and even the existence of a super-archaic ‘ghost’ population [2]. In addition, the availability of Neanderthal and Denisova DNA sequences provides a phylogenetic status and chronological depth that have significantly enhanced the understanding of gene variation in modern humans for phenotype, metabolic, and immune traits (reviewed in [3]).

Red cell blood groups are powerful anthropological markers. Phenotype and genotype geographical distribution mirrors past human migrations and natural selection [4–7] and comparison with primates makes it possible to evoke their evolutionary and migration trajectory with accuracy [8, 9]. Red cell blood groups are also crucial in medicine to ensure transfusion safety, transplants, and foeto-maternal compatibility [10]. To date, the International Society of Blood Transfusion (retrieved from the ISBT website, http://www.isbtweb.org) has recorded more than 380 blood group specificities grouped into 40 systems. In transfusion, it is routine practice to scrutinize six blood groups: ABO, Rh, Kell, Duffy, Kidd and MNS (reviewed in [10]). Curiously, despite their significance and the amount of available genotypic data on modern Humans that is continuously accumulating [11], almost no attention has been paid to these major red cell blood polymorphisms in palaeogenetic studies [12].

In the present study, we analyze Neanderthal and Denisovan blood groups in order to trace back the current human diversity and to discuss health aspects and vulnerabilities of archaic populations. For that purpose, we investigated the high-quality nuclear genomes previously published from three Neanderthals one Denisovan.

## Material and methods

### Selection criteria of the probands

To ensure genotype calling rate, consistency across individuals and phylogenetic positioning in relation to anatomically modern humans, we did not consider contaminated, admixed, low-depth and archaic genomes with abundant uncalled positions in the loci understudy. We hence retained only high-quality genomes from one Denisovan (Denisova 3) and three Neanderthal individuals i.e., Altaï Neanderthal (Denisova 5), Vindija 33.19, and Chagyrskaya 8 [13–16] (S1 Table). These four probands are representative of the two archaic human-related species that spanned over 50,000 years of the Late Pleistocene and across approximately 5,000 km of Eurasia.

### Presentation of the blood groups under study

We studied 7 blood group systems covering 11 genes: ABO including H system and Secretor status (ISBT 001 and 018, *ABO*, *FUT1 and FUT2* genes), Rh (ISBT 004, *RHD* and *RHCE* genes), Kell and the covalently linked Kx protein (ISBT 006, *KEL* and *XK* genes), Duffy (ISBT 008, *ACKR1* gene), Kidd (ISBT 009, *SLC14A1* gene), MNS (ISBT 002, *GYPB* gene), and Diego and its Band 3-Memphis variant (ISBT 010, *SLC4A1* gene) (S2 Table).

### Exploration procedure for blood group alleles

For the probands and blood groups under study, we downloaded the already published [13–16] and curated *.vcf and *.bam(.bai) chromosome files available at the Genome Projects website of the Max Planck Department of Evolutionary Genetics (https://www.eva.mpg.de/genetics/genome-projects.html, S1 Table). For genotype calling filters, see the Supplementary Information of [13–16] and the readme files at http://cdna.eva.mpg.de/neandertal/. Briefly, the filters included a coverage filter stratified by GC content, minimum coverage of 10, Heng Li’s Mappability 35, Mapping Quality (MQ) of 25, no tandem repeats and no indels.

Then, we briefly proceeded in a two-step screening of the blood group loci. First, we first gathered the genotypes at the key functional changes with depth, allele counts, quality and Phred scores probability using vcftools [17] (S2 Table, S1 File). Second, we browsed all exomes regions within the coding bounds (i.e. from the initiation ATG to the stop codons), in search for additional variation from hg19 (S3 Table, S1 File). While doing so, we paid specific attention to the following five points.

#### Consideration of the reference sequence

We aligned with the reference blood group gene sequences used by the ISBT against the GRCh37 (hg19) with nucleotide labelling according to the sense (5’-3’) strand (S2 Table). We noticed that for six loci we studied (*ABO*, *KEL*, *GYPB*, *RHCE*, *SCL4A1* and *FUT1*) the hg19 reference sequence opens by default onto the antisense strand (3’ → 5’) in the NCBI graphic window and the ancient genome browser, although their cDNA is conventionally the sense strand (5’ → 3’). This has two consequences: the chromosomal coordinates are decreasing as we progress throughout the coding strand (5’ → 3’) of these genes (from exon ’n’ to exon ’n+1’) and overall, nucleotides should be reversed-complemented.

#### ABO genotype calls

We inferred the *ABO* alleles according to the functional approach developed by [18, 19] for pure and chimeric A-B transferase cDNAs. In conformity with this approach, we identified the *ABO* alleles by 4 letters corresponding to the 4 main amino acid changes in the catalytic site of the glycosyltransferase of pure A or B allele positions, preceded by the presence or not of the G in position c.261 (rs8176719) (i.e. G-AAAA meaning 4 SNPs of *A* allele generating A phenotype) and the deletion or not of the C at position c.1061 (rs56392308) to differentiate the *A1* and *A2* alleles. We achieved the *ABO* allele identification with the screening of all exons and collected the genotypes at 39 additional loci previously identified as responsible for various *ABO* alleles [20] (S2 Table; Fig A in S1 File). Special attention has been taken to *FUT2* whose amino acid numbering in NBCI and hg19 should be rectified to get the correct amino-acid changes as mentioned by the ISBT. This is due to the fact that the initiation codon is the third ATG at the beginning of exon 2 (19: 49,206,247) [21].

#### *RHD* and *RHCE* genotype calls

For *RHD* and *RCE*, while browsing the exons in search for variation with hg19, we gathered the genotypes of the key changes of the RH*Ce, *CE, *ce, and *cE alleles. Any variation with hg19 was consolidated with The Human Rhesusbase.com [22], Erythrogene.com [11] and screenshots of the bam sequences (Figs B-D in S1 File).

For any identification of a variant, we searched for it in all four archaic genomes. In addition, for any call at two key variants of our findings, namely c.733G>C (*RHD*) and c.712A>G (*RHCE*), we searched for the other polymorphisms that usually constitute the haplotypes made with them, respectively RHD*DBU, *DLX, *DV, *DVI, *DBS, *DBT, *DUC2 and *ceAR, *ceEK, *ceBI, *ce*SM, and *ceHAR. For this, we browsed both vcf and bam alignment by varying the MQ threshold (S2 Table; Fig D in S1 File).

#### Identification of indels

Because indels could have been filtered out in the making the vcf files, all *ABO*, *RHD* and *RHCE*, notably the *ABO* c.261delG, c.1061delC, and *RHCE* 209bp insert have been double-checked from the specific indels vcf files (http://ftp.eva.mpg.de/neandertal/Vindija/VCF/indels/) and bam alignments using Integrative Genomics Viewer (IGV [23]) (S2 Table and Fig A in S1 File).

#### Low-mapped variants

The screenshots of the bam alignments in simultaneously the four archaic individuals have highlighted a difference in depth and MQ between reference and alternate alleles. This is especially true for variants with very low frequency in modern humans reference panel such as rs17418085 (*RHD*), rs150073306 (*RHD*), rs1132763 (*RHCE*), and rs1132764 (*RHCE*) in the 3 Neandertals (alternate allele) in comparison with Denisova 3, homozygous for the reference alleles (Figs C and D in S1 File). Hence, these loci may suffer from reference bias, which is known to strongly reduce the depth and mapping of the reads with the alternate alleles [24, 25], and consequently, the genotype accuracy indexes at these loci. Hence, in cases where variants have been called in the released VCFs of some probands but filtered out in the others, we screenshotted the indexed alignments with hg19 using IGV [23] to manually call genotypes with allocated reads count and MQ cut-off (i.e. value above which the reads are not visualized) (S1 File).

## Results

**Fig 1:**
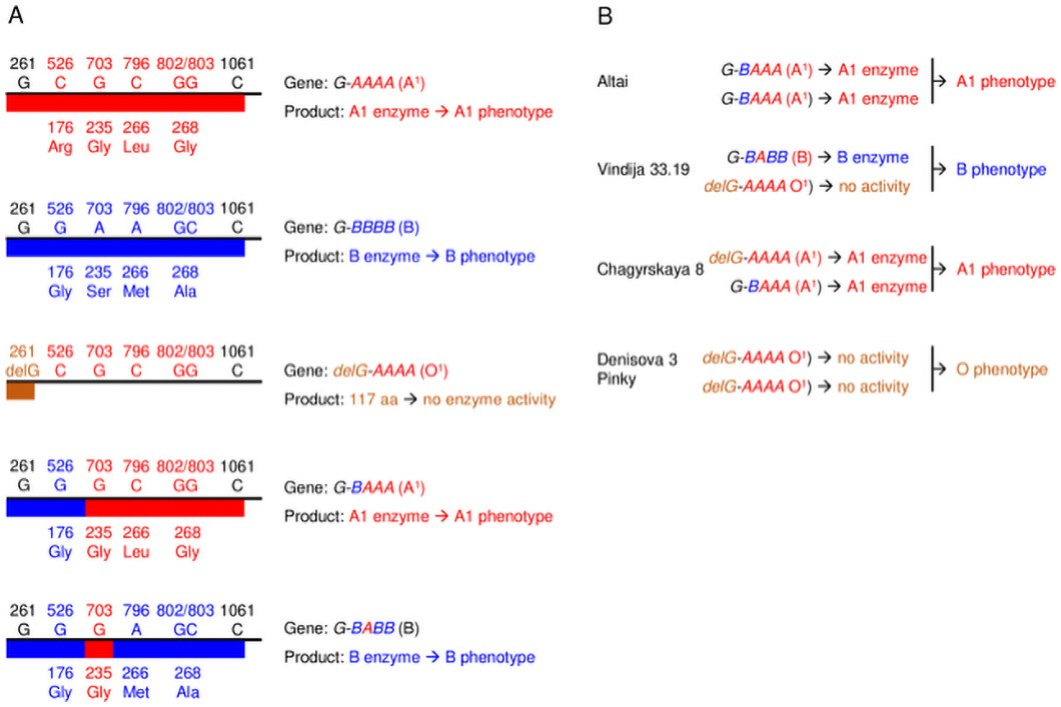
ABO system. A, Nomenclature of the archaic *ABO* alleles [18]. B, Different observed genotypes and inferred phenotypes in Neandertal and Denisova. Red: polymorphisms characteristic of the ABO*A allele, blue: polymorphisms characteristic of the ABO*B allele, brown: deletion characteristic of the ABO*O01 allele.

**Fig 2:**
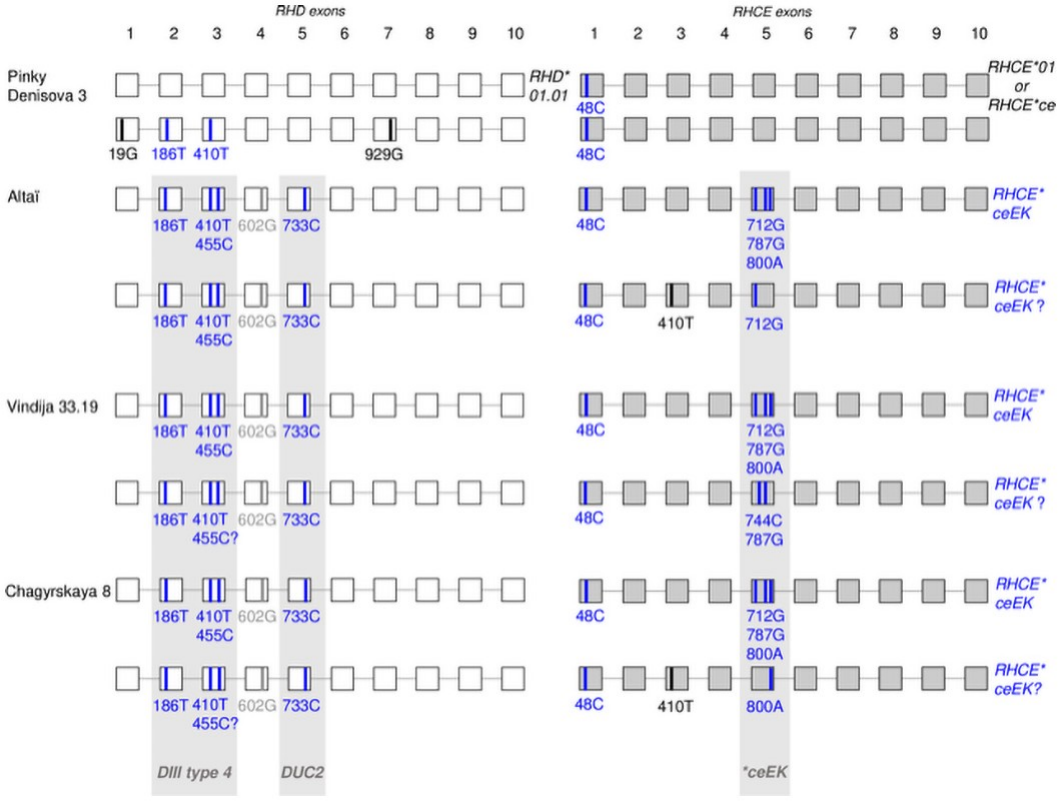
Representation of the different *RHD* and *RHCE* genotypes in Neandertal and Denisova. Black font, unknown allees in modern humans; blue font, known alleles in modern humans; gray shadow, allele background found in modern humans.

Detailed information for the blood group systems, genotypes and phenotypes as well as for other polymorphisms identified in these archaic hominins is presented in Tables 1 and 2 and the principal information is shown in Figs 1 and 2.

**Table 1:** Blood group systems, genotypes and phenotypes of four Denisova and Neanderthal archaic genomes. Each occurrence of a SNP is scored by the "+" character.

System, ISBT	Genotype: nt, aa	Phenotype	cDNA							
			hg19		NCBI		"Pinky" Denisova3	Altai	Vindija 33.19	Chagyrskaya 8
ABO, 001	c.261G	A1, A2, B, O2	uc004cda.1 (no-patched)		NM_020469.3	+		+	+	+
	c.261G, p.88fs118stop	O1		+			+		+	+
	c.526C, p.176Arg	A1, A2, O1		+		+	+		+	+
	c.526G, p.176Gly	B, O2						+	+	+
	c.703G, p.235Gly	A1, A2, O1, O2		+		+	+	+	+	+
	c.703A, p.235Ser	B								
	c.796C, p.296Leu	A1, A2, O1, O2		+		+	+	+	+	+
	c.796A, p.296Met	B							+	
	c.802/3GG, p268Gly	A1, A2, O1		+		+	+	+	+	+
	c.802/3GC, p268Ala	B							+	
	c.802/3AG, p268Arg	O2								
	c.1061delC, p.Pro354Argfs*21	A2								
	**Genotype**		*delG*. AAAA ABO*O.*01*.*01*		G-AAAA ABO*A1.*01*		*delG- AAAA / delG- AAAA*	*G-BAAA / G-BAAA*	*delG*. *AAAA / G-BABB*	*delG-AAAA / G-BAAA*
		**Phenotype**	O^1^		A^1^		O^1^	A^1^	B	A^1^
H, 018	FUT1*01/*01: c.35C, p.12Ala	H+	uc002pkk.3	+	NM_000148.3	+	+	+	+	+
	FUT2*01/*01: c.4G, p.2Ala	Se	uc002pke.4 (short)	+	MN_000511.5 (short)	+	+	+	+	+
	c.571C, p.191Arg	Se		+		+	+	+	+	
	FUT2*01N.*04*: c.571T, p.191stop	se							+	+
	c.714T, p.238Cys	Se		+		+	+	+	+	+
	FUT2*01N: c.714A, p.238stop	se					+			
	**Genotype**		*HH*, *SeSe*		*HH*, *SeSe*		*HH*, *Sese &*	*HH*, *SeSe*	*HH*, *Sese*	*HH*, *sese*
MNS, 002	GYPB*04/*04: c.143C, p. 48Thr	s	uc003ijm.1	+	NM_002100.4	+	+	+	+	+
	c.251C, p.84Thr					+				
	c.251G, p.84Ser			+			+	+	+	+
RHD, 004	c.186G, p.62Leu	RhD	uc001bjz.3	+	MN_016124.3	+	+			
	c.186T, p.62Phe						+	+	+	+
	c.410C p.137Ala	RhD		+		+	+			
	c.410T, p.137Val						+	+	+	+
	c.455A, p.152Asn	RhD		+		+	+			
	c.455C, p.152Thr							+	+	+
	c.602C, p.201Thr	RhD		+		+	+			
	c.602G, p.201Arg							+	+	+
	c.733G, p.245Val	RhD		+		+	+			
	c.733C, p.245Leu							+	+	+
	c.929A, p.310Lys	RhD		+		+	+	+	+	+
	c.929G, p.310Arg						+			
	**Genotype**		RHD*01		RHD*01		RHD*01/RHD*DIII type4(929G and without 455C)	RHD*DIII type4 (602G and 733C)/ RHD*DIII type4 (602G and 733C)	RHD*DIII type4 (602G and 733C)/RHD*DIII type4 (602G and *733C)*	RHD*DIII type4 (602G and 733C)/ RHD*DIII type4 (602G and *733C)*
		**Phenotype**	RhD		RhD		RhD	probably partial RhD	probably partial RhD	probably partial RhD
RHCE, 004	c.48G, p.16Trp	Rhce, RhcE	uc001bkf.03		NM_020485.4	+				
	c.48C, p16cys	RhCE, RhCe		+			+	+	+	+
	c.178A, p.60Ile	RhCE, RhCe								
	c.178C, p60Leu	RhcE, Rhce		+		+	+	+	+	+
	c.203G, p.68Ser	RhCE, RhCe								
	c.203A, p.68Asn	RhcE, Rhce		+		+	+	+	+	+
	c.307T, p.103Ser	RhCE, RhCe								
	c.307C, p.103Pro	RhcE, Rhce		+		+	+	+	+	+
	IVS2.ins+109bp	RhC								
	c.676C, p.226Pro	RhCE, RhcE								
	c.676G, p.226Ala	RhCe, Rhce		+		+	+	+	+	+
	c.410T, p.137Val	new variant						+		+
	c.410C, p.137Ala	RhCE, Ce, ce, cE		+		+	+	+	+	+
	c.712G, p.238Val	partial and weak antigens						+	+	+
	c.712A, p.238Met	RhCE, Ce, ce, cE		+		+	+		+	+
	c.744C, p.248Ser	partial and weak antigens							+	
	c.744T, p.248Ser	RhCE, Ce, ce, cE		+		+	+	+	+	+
	c.787G, p.263Gly	partial and weak antigens						+	+	+
	c.787A, p.263Arg	RhCE, Ce, ce, cE		+		+	+	+		+
	c.800A, p.267Lys	partial and weak antigens						+	+	+
	c.800T, p.267Met	RhCE, Ce, ce, cE		+		+	+	+	+	
	**Genotype**		RhCE*ce(48C)		RhCE*ce		RhCE*ce(48) / *ce(48C, *410T)*	RHCE*ceEK / *ce(48C, *410T*, *712G)*	RHCE*ceEK / *ce(48C, *744C*, *787G)*	RHCE*ceEK / *ce(48C, *410T*, *800A)*
		**Phenotype**	Rhce		Rhce		Rhce	partial Rhce	partial Rhce	partial Rhce
KEL, 006	KEL*02/*02: c.578C, p.193Thr	k	uc003wcb.3	+	NM_000420.2	+	+	+	+	+
	KEL*07/*07: c.1790T, p.597Leu	Js^b^		+		+	+			
	KEL*06/*06: c.1790C, p.597Pro	Js^a^						+	+	+
FY, 008	FY*01/*01: c.125G, p.42Gly, c.-67t	Fy^a^	uc001fto.3	+	NM_002036.3	+				
	FY*02/*02: c.125A, p.42Asp, c.-67t	Fy^b^					+	+	+	+
JK, 009	JK*01/*01: c.838G, p.280Asp	Jk^a^	uc010xcn.2	+	NM_015865.6	+	+	+	+	+
DI, 010	DI*02/*02: c.2561C, p.854Pro	Di^b^	uc002igf.4	+	NM_000342.3	+	+	+	+	+
	c.166G, p.56Glu						+		+	+
	c.166A, p.56Lys			+		+		+	+	+

**Table 2:** Other polymorphisms identified in blood group genes from Denisova and Neanderthals.

System, ISBT	Chr	rs number	nt, aa	cDNA							
				hg19		NCBI		"Pinky" Denisova3	Altai	Vindija 33.19	Chagyrskaya 8
ABO, 001	9	rs782228072	c.43T, p.15Tyr	uc004cda.1 (no-patched)		NM_020469.3		+			
			c.43C, p.15His		+		+	+	+	+	+
			c.488C, p.163Thr		+		+	+	+	+	+
		rs55756402	c.488T, p.163Met					+			
		rs782713806	c.777T, p.259Asp					+			
			c.777C, p.259Asp		+		+		+	+	+
		rs578090581	c.799C, p.267Arg					+			
			c.799G, p.267Gly		+		+		+	+	+
KEL, 006	7	no rs	c.657G, p.219His	uc003wcb.3	+	NM_000420.2	+		+	+	+
			c.657A, p.219His					+			
		rs139319150	c.936C, p.312Pro		+		+	+	+	+	
			c.936T, p.312Pro								+
		rs370938244	c.1963C, p.655Arg		+		+	+	+	+	+
			c.1963T, p.655Trp					+			
		no rs	c.1967G, p.656Arg					+			
			c.1967A, p.656His		+		+		+	+	+
JK, 009	18	rs11357896	c.196A, p.Met66	uc010xcn.2		NM_015865.6		+			
			c.196G, p.66Val		+		+		+	+	+
		rs113029149	c.394A, p.Ile132					+			
			c.394G, p.132Val		+		+		+	+	+
		rs28994287	c.471A, p.157Val					+			
			c.471G, p.157Val		+		+		+	+	+
		rs2298718	c.756G, p.252Pro					+			
			c.756A, p.252Pro		+		+		+	+	+
DI, 010	17	rs5015	c.1249T, p.417Leu	uc002igf.4		NM_000342.3			+	+	+
			c.1249C, p.417Leu		+		+	+		+	+
		rs148115666	c. 1272T, p.424Gly							+	+
			c. 1272C, p.424Gly		+		+	+	+	+	+
H, 018	19	no rs	c.717G, p.Ser239	uc002pkk.3	+	NM_000148.3	+	+	+	+	+
			c.717A, p.Ser239						+		
		rs180026	c.375G, p.Glu125	uc002pke.4 (short)		MN_000511.5 (short)		+	+	+	+
			c.375A, p.Glu125		+		+				
		rs370886251	c.400G, p.Val134		+		+	+	+	+	+
			c.400A, p.Ile134					+			
		rs752579948	c.487A, p.163Met								+
			c.487G, p.163Val		+		+	+	+	+	+
		rs1474796067	c.807G, p.269Glu					+			
			c.807C, p.269Gln		+		+	+	+	+	+
		rs485186	c.960G, p.320Thr						+	+	+
			c.960A, p.320Thr		+		+	+			+
RH, 004	1		c.19C, p.Arg7		+		+	+	+	+	+
		rs142037235	c.19G, p.Gly7					+			
KX, 019	X		c.72G, p.Leu24		+		+	+			
		rs1556440110	c.72A, p.Leu24						+	+	+

The results of the analysis for each blood group system can be resumed as follows:

*ABO system*. We found the most common phenotypes present in modern human populations: A1, B and O resulting from the combination of 3 different alleles (Fig 1). Two samples are heterozygous: Chagyrskaya 8 with group A1 (delG-AAAA/G-BAAA without C deletion at position 1061) and Vindija 33.19 with group B (delG-AAAA/G-BABB) and two are homozygous: Altaï with group A1 (G-BAAA/G-BAAA without C deletion at position 1061) and Denisova-3 with group O (delG-AAAA/delG-AAAA).

*H/Se system*. All the samples present a common *FUT1* allele. Concerning *FUT2* locus, 3 alleles are found: the conventional functional FUT2*01 allele and two non-functional alleles: FUT2*01N.*04* and FUT2*01N: c.714A, p.238stop. Although FUT2*01N.*04* (Neanderthal Vindija and Chagyrskaya) is presently found in South East Asian and Oceanian populations [26–28], the latter allele, present in a single dose in Denisova-3, has never been described in modern humans [10, 11].

*RH system*. All of the backgrounds are based on the *Dce (R*^*0*^*)* haplotype as described in Africa [8] and we found only one complete *Dce (R*^*0*^*)* haplotype corresponding to the wild type described in modern human populations [8]. This complete *R*^*0*^ haplotype is present, at a single dose, in the Denisova-3 sample. The other haplotypes are variants encoding partial antigens D, c and e, which are missing some epitopes. All of these haplotypes, whether wild or variant, are related, phylogenetically speaking, to the African RHD*DIVa cluster [8]. From the three Neanderthal genomes, we have identified one potential novel *RHD* variant sharing typical SNP combinations assigned to this cluster (Fig 2). This variant exhibited the three SNPs that characterize the partial RHD*DIII type4 (or RHD*03.*04* defined by c.186T, c.410T and c.455C), combined with two additional SNPs, c.602C>G and c.733G>C. This latter SNP defining the partial RHD*DUC2 allele. This new combination has been reported for the first time in only one modern Australian Aborigine [29] and occurred at a homozygous level in Altaï, and at least at a heterozygous level in Vindija and Chagyrskaya, with a normal *RHD* Exon-9 sequence (c.1170T, c.1193A) for all of them. This new RHD variants segregated with RHCE*ce allele variants although the RHD*DIII type4 has been reported to segregate with RHCE*Ce in modern humans [30].

Furthermore, we found the presence of at least three RHCE*ceEK variant alleles (c.48G>C, c.712A>G, c.787A>G and possibly c.800T>A) in Altai Neandertal, Vindija and Chagyrskaya, and two wild-type RHCE*ce in Denisova-3. Each of the Neanderthal archaic genomes has one other African RHCE*ce variant in cis. The RHCE*ceEK allele is mostly encountered in people of African descent [31]. When present in double dose, it encodes a rare phenotype defined by the absence of public antigen RH:-18. The anti-RH18 antibody has been reported to be responsible for haemolytic transfusion reactions and haemolytic disease of the foetus and new-born (HDFN) [32].

*MNS*, *KEL*, *Duffy*, *Kidd and Diego systems*. All four archaic samples present the deduced phenotype S-s+, K negative, Fy(a-b+), Jk (a+b-) and Di (a-b+) due to double-dose of MNS*04, KEL*02, FY*02, JK*01, DI*02 respectively. The three Neanderthals present the deduced phenotype Js(a+b-) of the Kell system due to double-dose of the ancestral KEL*06 allele, which is exclusive to African populations with 1% of Js(a+b-) phenotype [11]. On the contrary, Denisova 3 presents the deduced Js(a-b+) phenotype resulting from a double-dose of the antithetical KEL*07 allele. Lastly, the three Neanderthals are carriers of the Band 3-Memphis variant (according to rs5036).

## Discussion

Neanderthals are a human hunter-gatherer fossil population that lived in Eurasia between 250 kya and 38 kya before being totally replaced throughout their territory by *Homo sapiens*. Morphological features progressively evolved from their African ancestors (*Homo heidelbergensis*) and adapted to the cooler climate of Europe [33]. Their arrival in Europe marks a major cultural change with the importation of a new tool, well known in Africa since at least 1.5 mya and in the Levant, the handaxe [34]. The Denisovans are also an extinct human population but bone record is too fragmentary. Their "discovery" and phylogeny relies only on genetic studies [35, 36].

**Fig 3:**
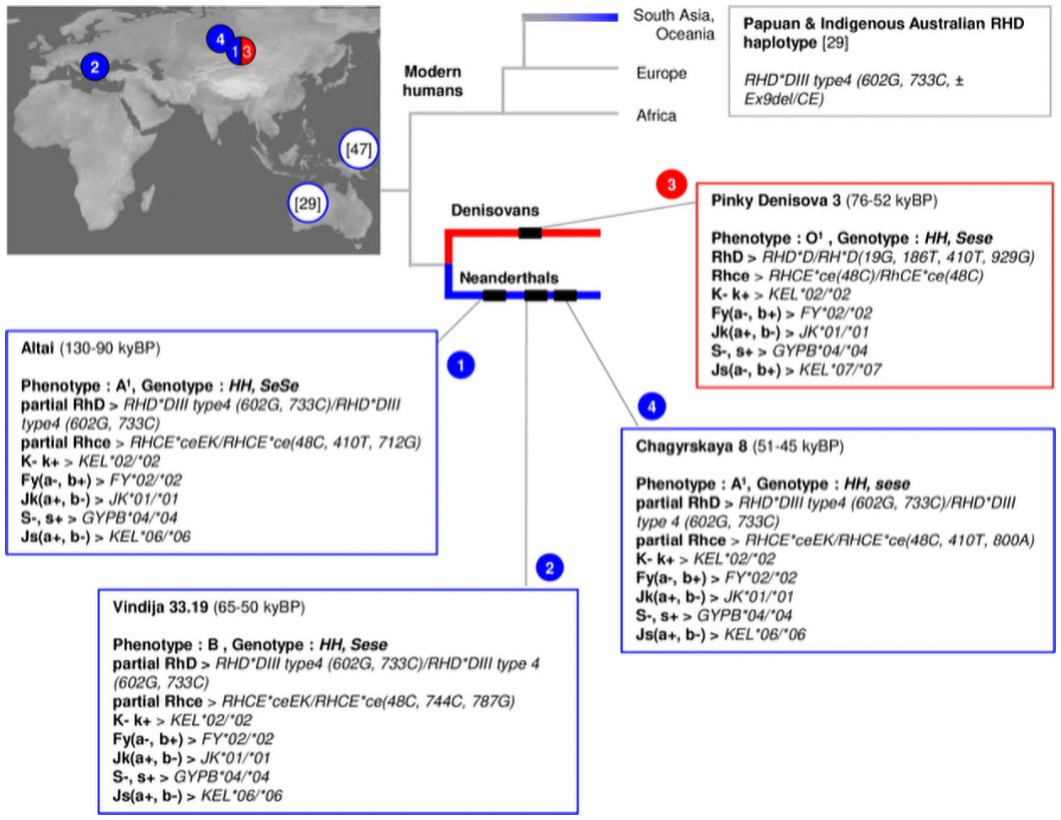
Erythroid blood group distribution from Denisova and Neanderthal archaic genomes. Branching matches nuclear DNA tree topology [43]. Blue, Neanderthal lineage; red, Denisovan lineage. Made with Natural Earth. Free vector and raster map data @ naturalearthdata.com.

**Fig 4:**
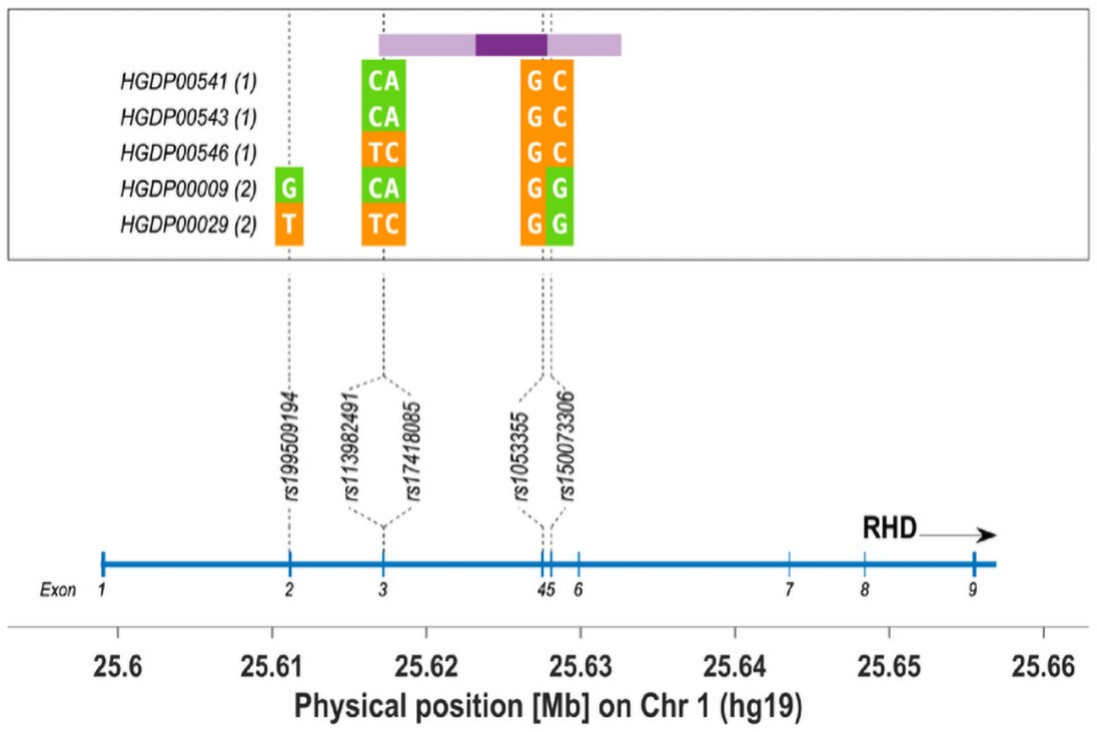
Introgression scan of modern humans in the *RHD* region. Introgression tracts were called at confidence > = 0.2 and <0.5 (15.7 Kb, light purple) and confidence > = 0.5 and <0.9 (4.6 Kb, dark purple) in 5 haplotypes from 2 HGDP-CEPH populations (1: Papuans Sepik; 2: Brahuis). Alleles carried by these haplotypes at 5 key SNPs for the RHD*DIII type 4 with c.602G and DUC2 are shown in green or orange, whether they are the ancestral or derived allele in the human lineage. The bottom panel shows the position of these SNPs on the *RHD* map.

Neanderthal populations were never large, structured in small interconnected groups (about 20 individuals) that never outnumbered 70,000 individuals at the time of their "golden age" (the "emian" interglacial at OIS 5, around 120 kya) [37]. The small size of the population has to be correlated with the partial geographic isolation of Neanderthals caused by European climatic fluctuations during Pleistocene. It was at this time that the Neanderthals, well identified by singular morphological features, spread eastwards carrying the same lithic assemblages and technology as far east as the Altai mountains [38] where they encountered the Denisovans. Genetic data has also pointed out the low genetic diversity of Neanderthals, with a demographic depression peak in the Altai where a very consanguineous individual was found [14], and genetic continuity across Europe from 120 kya until the disappearance of the population around 40 kya [39]. This low variability is also visible in the morphology of the Neanderthals, which remained the same during the last 100 kya of their existence throughout their territory from the Atlantic to the Altai [40]. In this view, our results provide four main points relevant for the origin, vulnerabilities and dispersion of Neanderthal and Denisova (Figs 3 and 4).

### Consolidation of an African origin for Neanderthal and Denisova

First, the analysis of archaic blood groups anchors the lineage of Eurasian Neanderthals and Denisovans to Africa via: the absence of the antigen combination RhC, RhE, K, Fy^a^, Jk^b^ and S (14% of sub-Saharan African populations, 0.06% in non-African populations [10]), the presence of a double-dose of ancestral forms of the RH, Kell, Duffy, Kidd, MNS and Diego blood groups (respectively the haplotype *Dce/Dce*, KEL*02/*02, KEL*06/*06, FY*02/*02, JK*01/*01, MNS*04/*04 and Memphis form of Band 3 system [8, 41, 42], and *RHD*, *RHCE* variants phylogenetically linked or presently exclusive to African clusters and populations [31]. These features are in accordance with a Neanderthal and Denisovan gene pool pre-dating the exit of *Homo sapiens* from Africa [43].

### A (mysterious) genetic link between Neanderthals and Australia

The new partial *RHD* allele, RHD*DIII type4 with c.602G and c.733C, reported in the three Neanderthal individuals, and not in Denisova 3, was unknown in modern humans until 2019 and its description as a new variant in an individual from the First Nation of Australia with a *RHD* Exon-9 deletion or rearrangement with *RHCE* Exon-9 [29]. Thus, this polymorphism is not a new variant in the historical sense of the term, as it was already present around 100 kya in Neanderthals. This result fuels the discussion about admixture events between the different lineages and also about the early dispersal of *Homo sapiens* via the Arabian Peninsula [44] towards Australia [45] and Oceania [46]. In order to assess whether the Neanderthal RHD*DIII type4 with c.602G and c.733C arose in modern humans from shared ancestry or introgression, we have modelled high-coverage modern chromosomes from Asia and Oceania (HGDP-CEPH collection, publicly available, [47]) as a mosaic of modern and archaic states using a hidden-Markov model (see S2 File for detailed methods). We found a probable introgressed tract of 15.7 Kb spanning over 4 exons of *RHD* in 5 individuals: 2 HGDP-Brahuis and 3 HGDP-Sepik Papuans (Fig 4). The 3 Sepik Papuans are heterozygous carriers of the derived allele at rs150073306 which defines the DUC2 allele (raising thus the MAF of the derived allele nearly to 19% in that population—see S2 File), for which Altai is homozygous. When phased, there is an almost identical haplotype to Altai and the Australian Aborigine in Papuan HGDP00546. A parsimonious hypothesis to explain the relic of a *RHD* haplotype common amongst all Neanderthals in 2 modern Oceanians only, would suggest that the RHD*DIII type4 with c.602G and c.733C profile have been carried by Levantine Neanderthals and passed to modern humans before 65 kya and the route towards SouthEast Asia [48, 49]. This assumption remains however speculative as the archaic tract was called at a low confidence level (>20%). A shorter tract (4.6Kb) was still called at a confidence level of >50% but not at higher confidence thresholds (>90%). Further analyses would be required to validate our assumption.

### The signature of environmental pressure

Our analysis of the *ABO* and *FUT2* genes supports the fact that pathogens have played a major role in the survival and selection of genetic variants in modern and archaic hominins [50]. The molecular bases of the archaic ABO phenotypes, particularly for A and B groups, are identical to those found in non-human primates, which confirms an ancient balanced selection shared by Primate species [51]. Noteworthy is the presence of two non-secretor *FUT2* polymorphisms in Neanderthal and Denisovan individuals. To date, the non-secretor phenotype has not been found in non-human primates [51] and shown to confer more resistance to some viruses with a notable resistance to Norovirus (Norwalk-like, *Caliciviridae* family) infection [52–54], which is a major cause of acute gastroenteritis worldwide. Given the short existence of the Norwalk-like virus in humans (post-Neolithic [55]), two scenarios would explain the occurrence of the non-secretor trait in archaic humans. *FUT2* could have evolved neutrally in hominins before the late occurrence of selective pressure, in the same way as for glucose transporters (GLUT 2 and 12) and G6PD2 with malaria [56]. Or, given that hominids likely hosted some viruses [57], a Norwalk-like virus (or a sister group) could have already existed 90,000 years ago across Eurasia, causing enough mortality in young children to select two convergent *FUT2* alleles. Note that the occurrence of the Neanderthal non-secretor allele in Oceanians [26–28] remains open.

### Demographic ‘fragility’

Lastly, our study highlights unfavorable characteristics that can lead to "demographic fragility". This fragility can be evoked on the basis of two elements: a low genetic diversity and the possible presence of HDFN. Indeed, the large number of shared alleles by the four archaic genomes despite their geographical and temporal distribution may be related to the deduced inbreeding situation in Neanderthals [14–16, 58], known to be a source of low adaptability. Meanwhile, the Neanderthal *RH* allele variants encode for partial RhD, Rhc and Rhe antigens, only Denisova 3 presents a complete form in terms of epitopes, such as they are described in their "wild" forms in modern humans. Partial RhD, Rhc and Rhe antigens lacking epitopes may induce an immune response when exposed to complete antigens [59, 60]. Moreover, when the RHCE*ceEK allele is present in a double dose (a situation which may turn out to be frequent in view of its presence in the 3 Neanderthals), in addition to the presence of partial Rhc and Rhe antigens, it encodes a phenotype defined by the absence of an Rh antigen named RH18. Today, this antigen is considered to be a high frequency antigen in the modern human population. Thus, a Neanderthal mother with partial RhD, Rhc, and Rhe phenotypes and sometimes RH:-18, carrying a Denisovan foetus expressing complete forms of RhD, Rhc and Rhe antigens and expressing the RH18 antigen, would have been prone to be immune to missing epitopes and synthesize anti-RhD, anti-Rhc, anti-Rhe and even anti-RH18 antibodies. These antibodies are known to have an important clinical significance in terms of HDFN [32]. These elements could have contributed to weakening the descendants to the point of leading to their demise especially combined with the competition with *Homo sapiens* for the same ecological niche [61].

## Conclusions

Analyses of blood group systems of Neanderthals and Denisovans contributed to a better understanding of their origin, expansion and encounters with *Homo sapiens*. Blood group profiles revealed polymorphism at the ABO locus, ancestral and African-origin alleles, and a RH haplotype presently secluded in Oceania, plausible relic of introgression events into modern humans prior their expansion towards Southeast Asia. An additional contribution is the reduced variability of many alleles and the possible presence of haemolytic disease of the foetus and new-born, which reinforces the notion of high inbreeding, weak demography and endangered reproductive success of the late Neanderthals, giving to our species the great opportunity to spread throughout the world.

## Supporting information

S1 Fig (TIF)

S1 File This file contains 5 figures showing screenshots of bam sequence alignments of loci for which vcf data are not available for at least one individual. (PPTX)

S2 File Introgression scan of modern humans in *RHD* region. (DOCX)

S1 Table Information about the individual analyzed in this study. (DOCX)

S2 Table ISBT nomenclatures of the blood group systems presented in Table 1, with archaic genotypes and Phred-score posterior probability. REF: reference base, ALT: alternate base, GT: (unphased) genotype. DP: read depth, GQ: Genotype Quality, PL: phred-scaled genotype likelihoods, A:C:G:T: number of A,C,G,T bases, IR: Number of reads with InDel starting at this position, PP = Phred-scaled posterior probability for genotypes AA, CC, GG, TT, AC, AG, AT, CG, CT, GT, in this order; 0/0: homozygous reference allele, 1/1: homozygous alternate allele, 0/1: heterozygous. Blue font: ABO key functional changes [18].
(XLSX)

S3 Table Genomic information and genotype posterior probability of the other red cell blood group polymorphisms observed in the Denisovan and Neanderthal individuals. REF: reference base, ALT: alternate base, GT: (unphased) genotype. DP: read depth, GQ: Genotype Quality, PL: phred-scaled genotype likelihoods, A:C:G:T: number of A,C,G,T bases, IR: Number of reads with InDel starting at this position, PP = Phred-scaled posterior probability for genotypes AA, CC, GG, TT, AC, AG, AT, CG, CT, GT, in this order; 0/0: homozygous reference allele, 1/1: homozygous alternate allele, 0/1: heterozygous.
(XLSX)
