# Supplementary figures and images for: Blood groups of Neandertals and Denisova decrypted
Source: PLoS One. 2021 Jul 28;16(7):e0254175. doi: 10.1371/journal.pone.0254175 (PMC8318287; doi:10.1371/journal.pone.0254175)

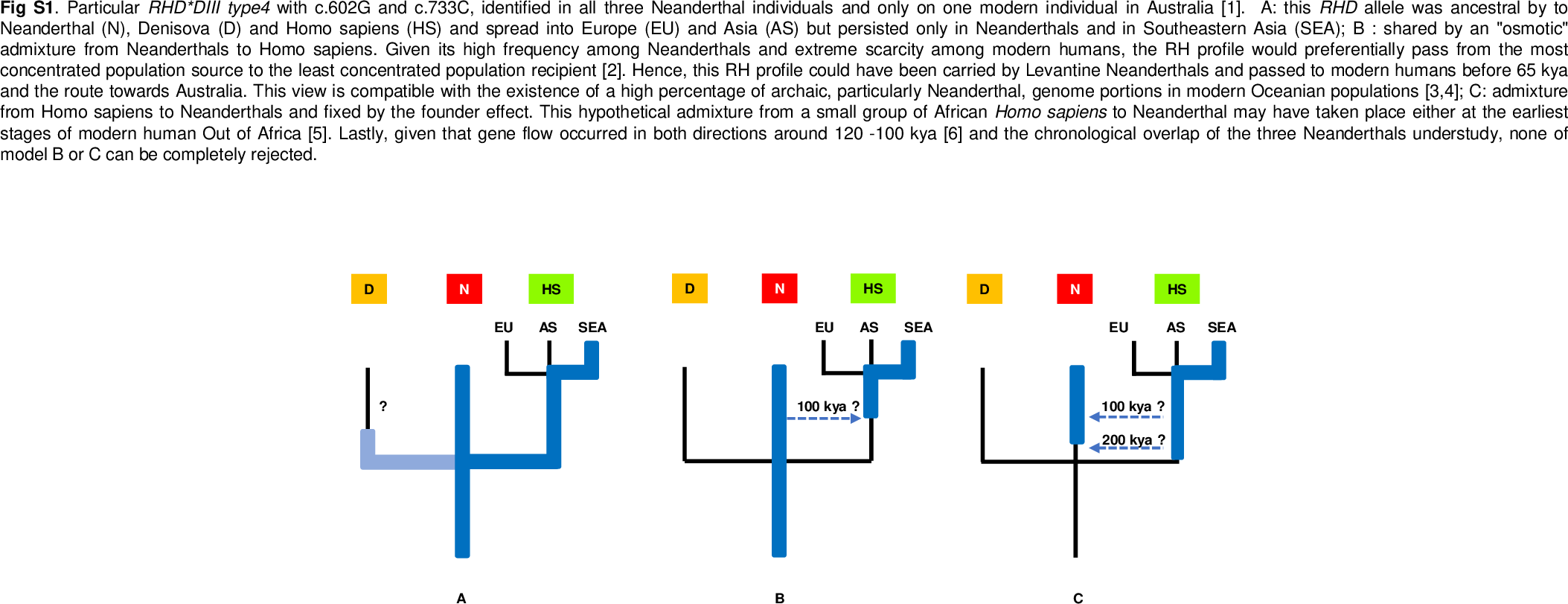

Supplement: S1 Fig — (TIF) [file pone.0254175.s001.tif]
