# Supplementary material for: Blood groups of Neandertals and Denisova decrypted
Source: PLoS One. 2021 Jul 28;16(7):e0254175. doi: 10.1371/journal.pone.0254175 (PMC8318287; doi:10.1371/journal.pone.0254175)
Supplement: S1 File — (PPTX) [file pone.0254175.s002.pptx]

## Slide 1
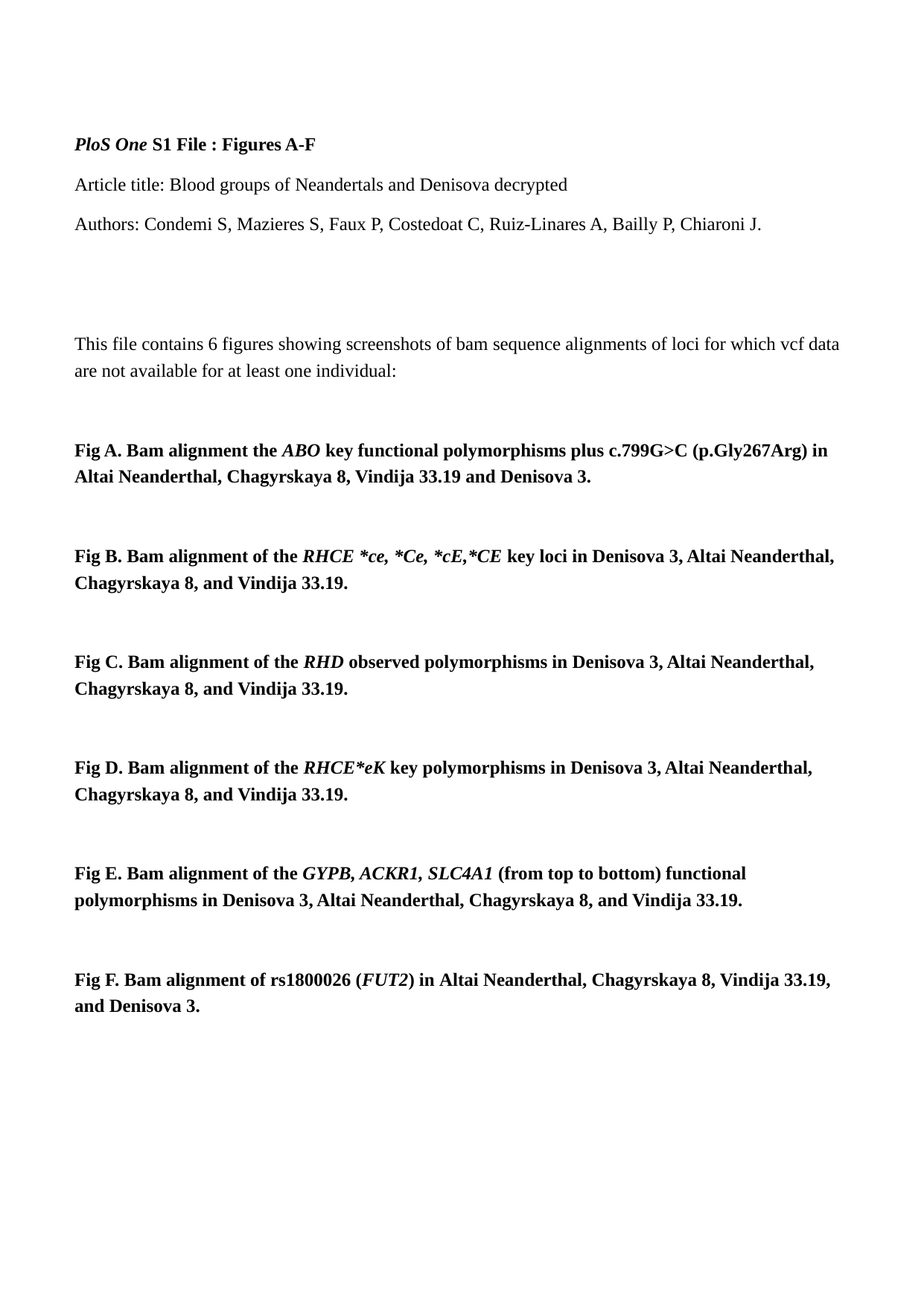

# PloS One S1 File : Figures A-F Article title: Blood groups of Neandertals and Denisova decrypted Authors: Condemi S, Mazieres S, Faux P, Costedoat C, Ruiz-Linares A, Bailly P, Chiaroni J.
This file contains 6 figures showing screenshots of bam sequence alignments of loci for which vcf data are not available for at least one individual:
Fig A. Bam alignment the ABO key functional polymorphisms plus c.799G>C (p.Gly267Arg) in Altai Neanderthal, Chagyrskaya 8, Vindija 33.19 and Denisova 3.
Fig B. Bam alignment of the RHCE *ce, *Ce, *cE,*CE key loci in Denisova 3, Altai Neanderthal, Chagyrskaya 8, and Vindija 33.19.
Fig C. Bam alignment of the RHD observed polymorphisms in Denisova 3, Altai Neanderthal, Chagyrskaya 8, and Vindija 33.19.
Fig D. Bam alignment of the RHCE*eK key polymorphisms in Denisova 3, Altai Neanderthal, Chagyrskaya 8, and Vindija 33.19.
Fig E. Bam alignment of the GYPB, ACKR1, SLC4A1 (from top to bottom) functional polymorphisms in Denisova 3, Altai Neanderthal, Chagyrskaya 8, and Vindija 33.19.
Fig F. Bam alignment of rs1800026 (FUT2) in Altai Neanderthal, Chagyrskaya 8, Vindija 33.19, and Denisova 3.

## Slide 2
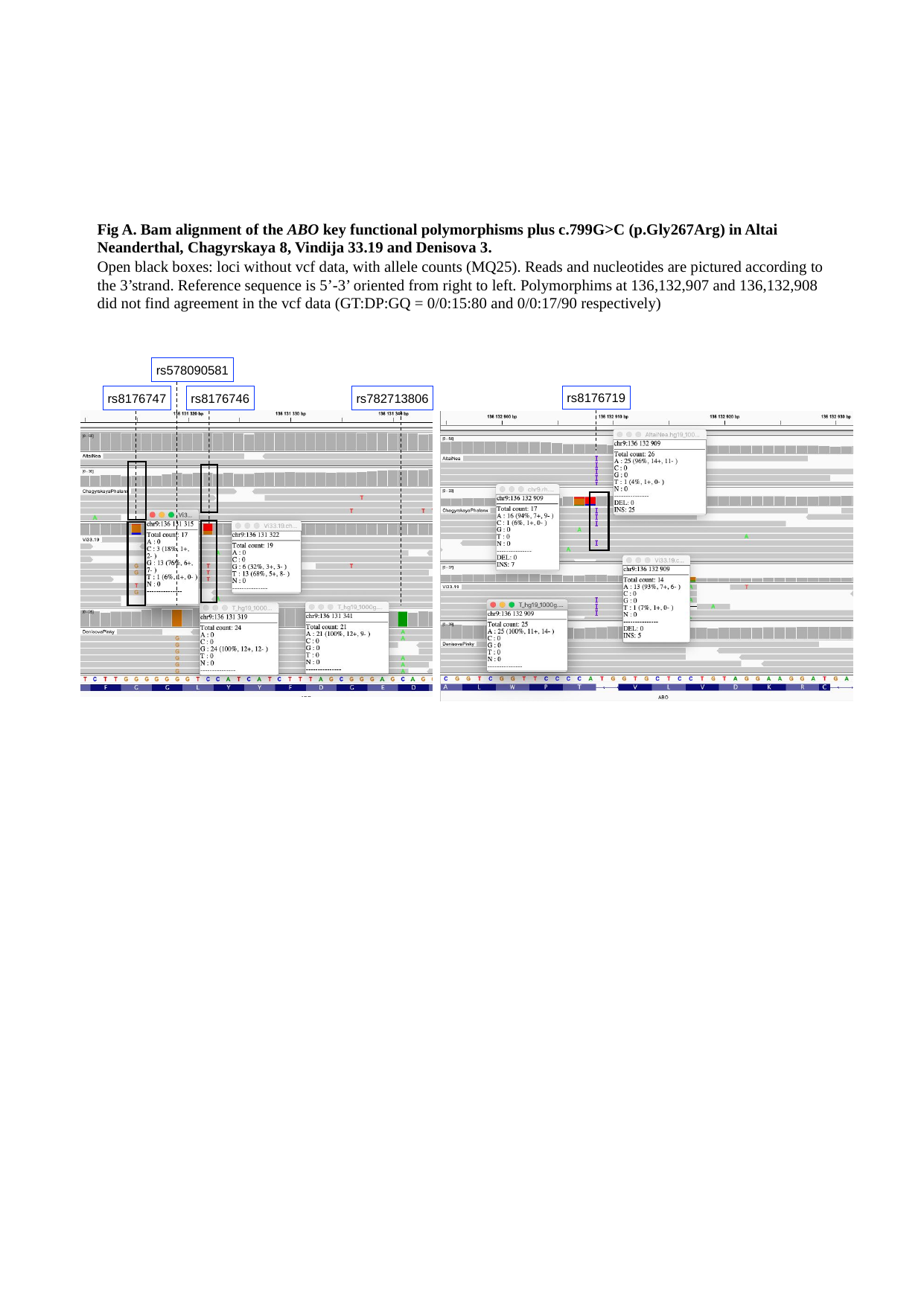

Fig A. Bam alignment of the ABO key functional polymorphisms plus c.799G>C (p.Gly267Arg) in Altai Neanderthal, Chagyrskaya 8, Vindija 33.19 and Denisova 3.
Open black boxes: loci without vcf data, with allele counts (MQ25). Reads and nucleotides are pictured according to the 3’strand. Reference sequence is 5’-3’ oriented from right to left. Polymorphims at 136,132,907 and 136,132,908 did not find agreement in the vcf data (GT:DP:GQ = 0/0:15:80 and 0/0:17/90 respectively)
rs578090581
rs8176719
rs8176747
rs8176746
rs782713806

## Slide 3
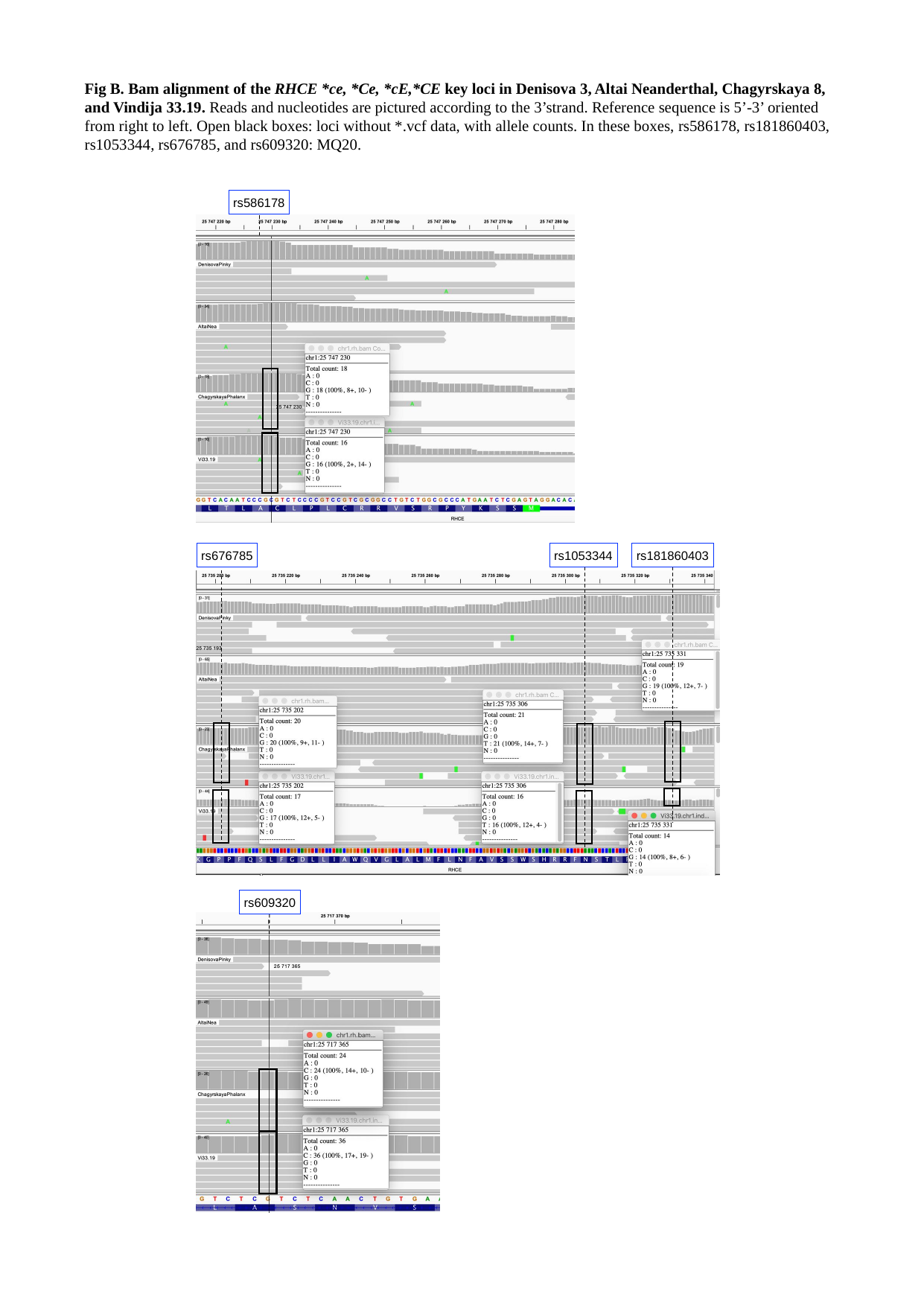

Fig B. Bam alignment of the RHCE *ce, *Ce, *cE,*CE key loci in Denisova 3, Altai Neanderthal, Chagyrskaya 8, and Vindija 33.19. Reads and nucleotides are pictured according to the 3’strand. Reference sequence is 5’-3’ oriented from right to left. Open black boxes: loci without *.vcf data, with allele counts. In these boxes, rs586178, rs181860403, rs1053344, rs676785, and rs609320: MQ20.
rs586178
rs676785
rs1053344
rs181860403
rs609320

## Slide 4
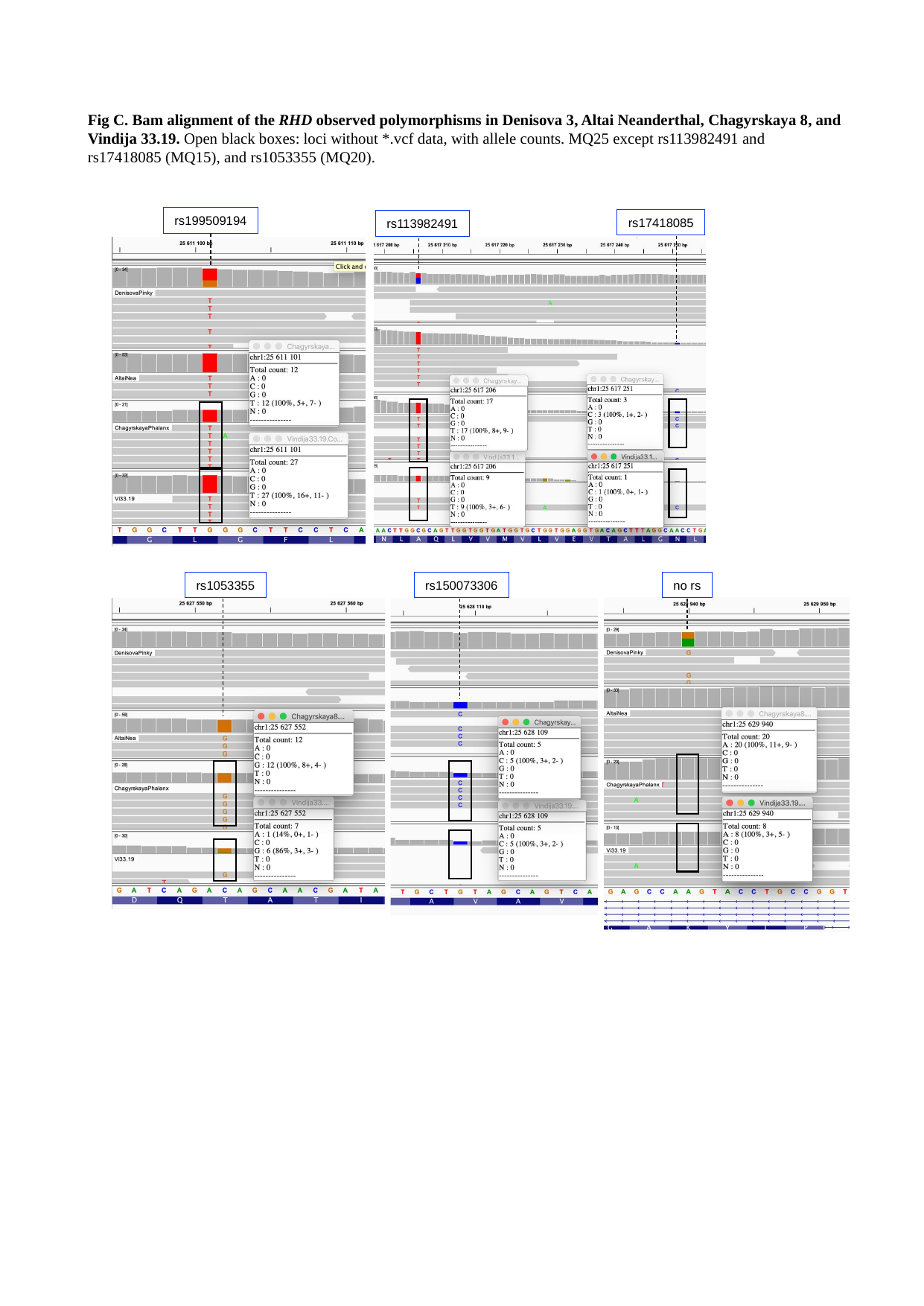

Fig C. Bam alignment of the RHD observed polymorphisms in Denisova 3, Altai Neanderthal, Chagyrskaya 8, and Vindija 33.19. Open black boxes: loci without *.vcf data, with allele counts. MQ25 except rs113982491 and rs17418085 (MQ15), and rs1053355 (MQ20).
rs199509194
rs17418085
rs113982491
rs1053355
rs150073306
no rs

## Slide 5
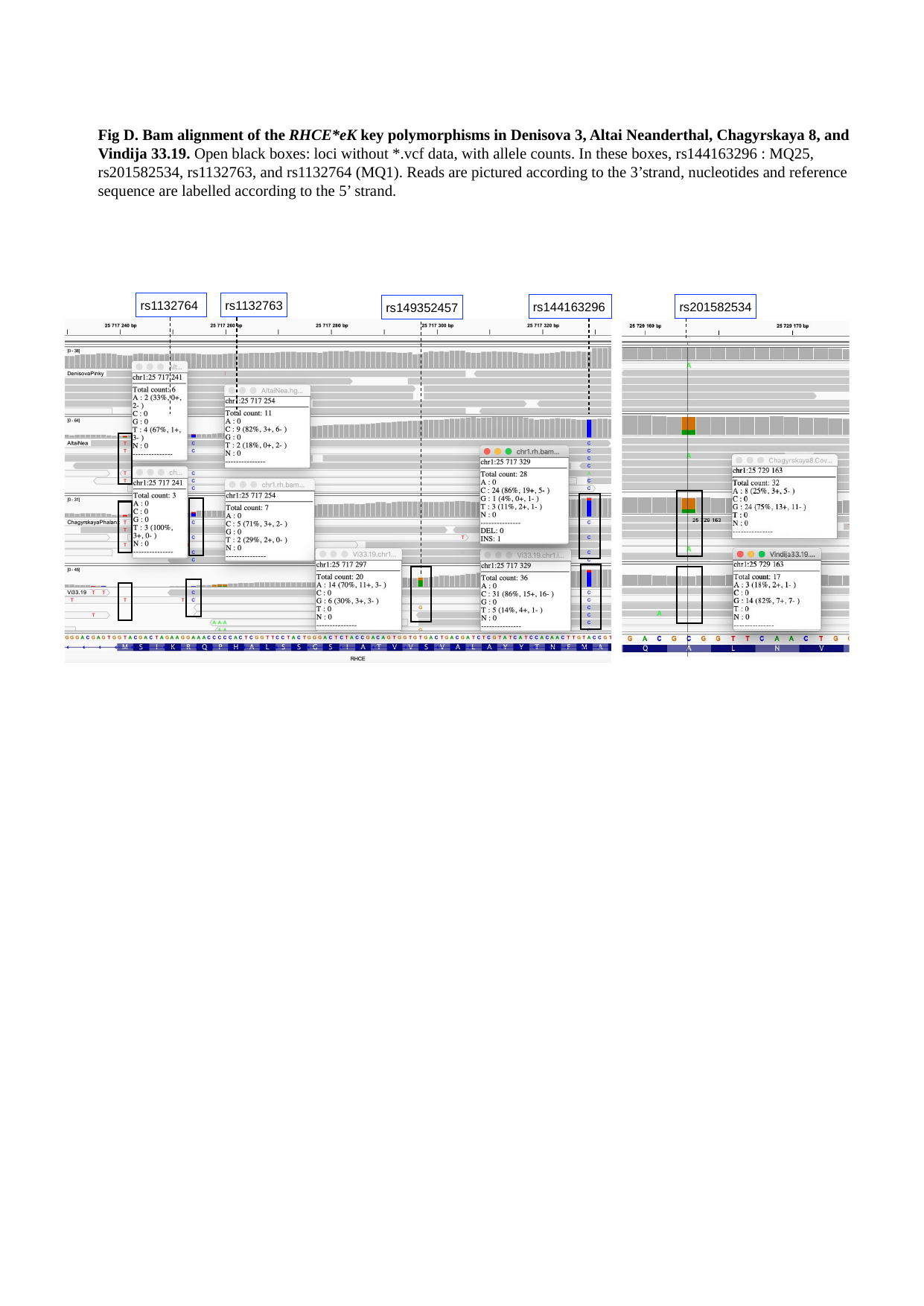

Fig D. Bam alignment of the RHCE*eK key polymorphisms in Denisova 3, Altai Neanderthal, Chagyrskaya 8, and Vindija 33.19. Open black boxes: loci without *.vcf data, with allele counts. In these boxes, rs144163296 : MQ25, rs201582534, rs1132763, and rs1132764 (MQ1). Reads are pictured according to the 3’strand, nucleotides and reference sequence are labelled according to the 5’ strand.
rs1132764
rs1132763
rs144163296
rs201582534
rs149352457

## Slide 6
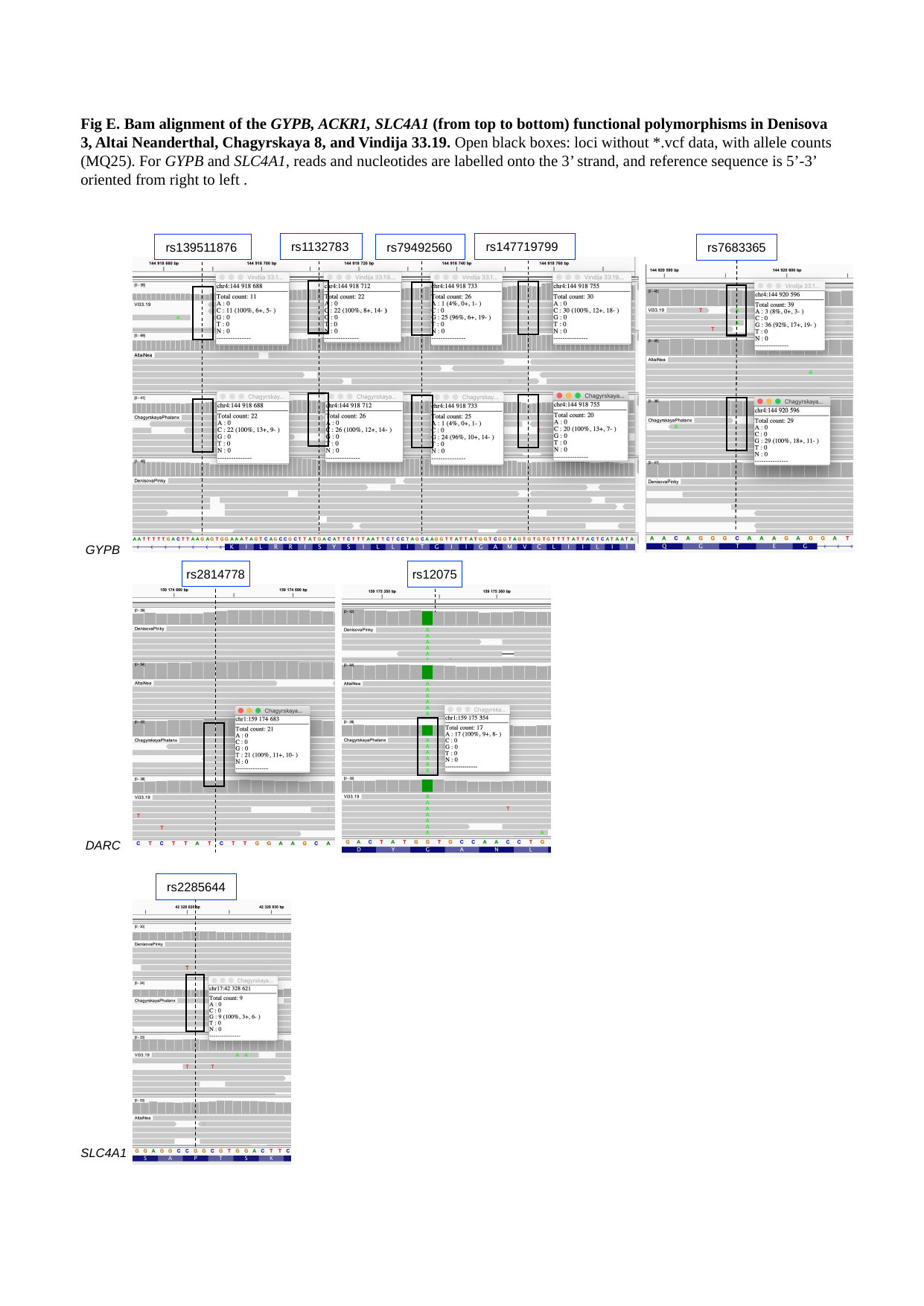

Fig E. Bam alignment of the GYPB, ACKR1, SLC4A1 (from top to bottom) functional polymorphisms in Denisova 3, Altai Neanderthal, Chagyrskaya 8, and Vindija 33.19. Open black boxes: loci without *.vcf data, with allele counts (MQ25). For GYPB and SLC4A1, reads and nucleotides are labelled onto the 3’ strand, and reference sequence is 5’-3’ oriented from right to left .
rs1132783
rs147719799
rs139511876
rs79492560
rs7683365
GYPB
rs2814778
rs12075
DARC
rs2285644
SLC4A1

## Slide 7
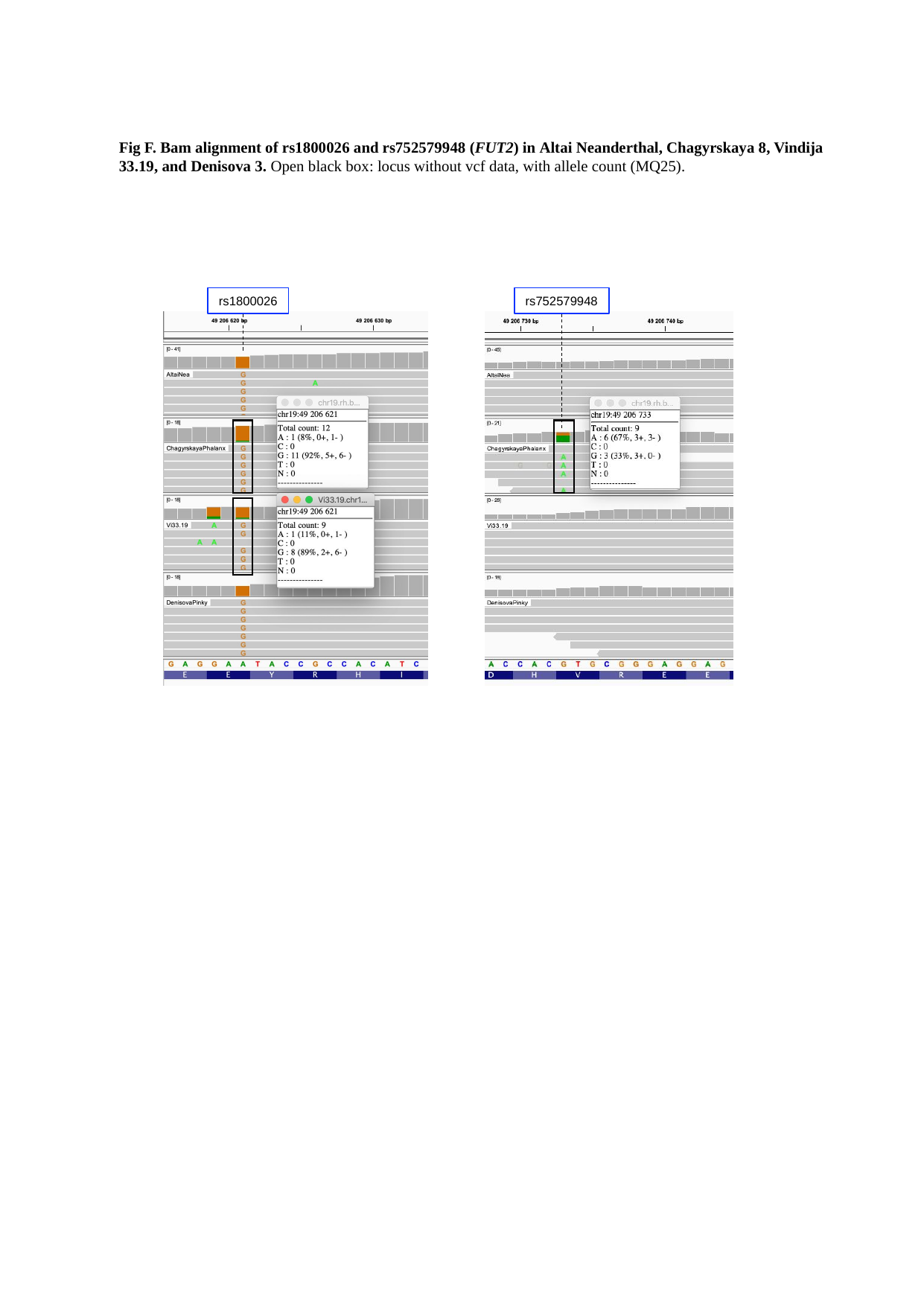

Fig F. Bam alignment of rs1800026 and rs752579948 (FUT2) in Altai Neanderthal, Chagyrskaya 8, Vindija 33.19, and Denisova 3. Open black box: locus without vcf data, with allele count (MQ25).
rs1800026
rs752579948
