# Supplementary material for: Blood groups of Neandertals and Denisova decrypted
Source: PLoS One. 2021 Jul 28;16(7):e0254175. doi: 10.1371/journal.pone.0254175 (PMC8318287; doi:10.1371/journal.pone.0254175)
Supplement: S2 File — (DOCX) [file pone.0254175.s003.docx]

# S2 File. Introgression scans in RHD region

In order to assess if the Neanderthal RHD allele (DUC2) is present in modern humans as a result of an archaic introgression, we have modelled modern chromosomes from Asia and Oceania as a mosaic of modern and archaic states. In this Supplementary Note, we outline the material and methods used to that end and provide details on the results of this analysis.

## Material and methods

We have retained for analyses the 420 Asian and 28 Oceanian individuals among the 929 individuals from the Human Genome Diversity Project – Centre d’Etude du Polymorphisme Humain (HGDP-CEPH) panel [1]. For all these 448 individuals, we phased the *RHD* gene region defined as *RHD* gene limits released in GENCODE v34 (2020-03-24) -/+ 2Mb flanks using Shapeit4 [2], yielding a total of 896 haplotypes. Only biallelic markers with mean coverage >20, call rate >90% and MAF>0.2% were kept for phasing. These filtering rules led to retain 4 of the 5 SNPs defining the Neanderthal haplotype made of the combination of DIII type 4 with c.602G and DUC2 alleles (rs113982491, rs17418085, rs1053355 and rs150073306). Discarded on call rate rule, rs199509194 was eventually pooled in the final selection of SNPs, leading to a total of 22,833 SNPs.

On the phased haplotypes, a subregion of 700Kb (from Hg37 physical coordinates 1:25,300,000 to 1:26,000,000) was further scanned for archaic introgression using *admixtureHMM* [3]. In brief, this program models the target haplotypes as a sequence of modern and archaic hidden states using modern and archaic frequencies of the alleles derived in the human lineage as references. For our analysis, we used (1) the YRI population from the 1000G Project Phase III (Yorubas in Ibadan, Nigeria - 108 individuals) as the modern reference and (2) the Altai Neanderthal sample [4] as the archaic reference. We retained 2,193 biallelic SNPs after merging on positions the markers that complied with the following rules in the 2 source populations (Asian and Oceanian individuals from HGDP-CEPH; YRI from 1000G Project Phase III) and the Altai Neanderthal sample: (a) mean coverage >20, (b) no allele discordance between the 3 sources, (c) no allele discordance with the ancestral allele reported in the Altai Neanderthal VCF (based on Chimp-Human comparison).

Both reference (frequencies) and target (haplotypes) data were aligned to the derived allele and we ran admixtureHMM with all default parameters except for confidence of call, which was set to 0.2, 0.5 and 0.9 in 3 separate runs.

## Results

### RHD haplotypes

The derived C allele of SNP rs150073306 (c.733G>C), which defines the DUC2 allele, has a frequency of 0.4% in the retained HGDP-CEPH individuals; all the 3 occurrences of that allele were actually sequenced in the “Papuan Sepik” group, a sub-population of 16 individuals from the Northern Lowlands of New Guinea [1], indicating a high frequency of that allele in that population (18.8%). One individual (HGDP00546, see Fig 4) among the three carriers was also carrying derived alleles at 3 other key SNPs on the same haplotype. Although not sequenced at the first key SNP for the DIII type 4-c.602G-DUC2 allele (rs199509194), that individual had read depths of 44 and 29 for the ancestral and derived alleles respectively, and rs199509194 is almost exclusively associated with rs113982491 and rs17418085 in modern humans [5], hence suggesting that he may carry the complete DIII type 4-c.602G-DUC2 haplotype as do the Neanderthal samples. The low MAFs of these SNPs in the phased population (from 0.2% to 0.6%) may however result in poor statistical phasing of these genotypes and should therefore caution this result.

### Introgression tracts

One archaic introgression tract of 15.7 Kb was found at a confidence level greater or equal to 20% in the neighborhood of the 5 key SNPs for the DIII type 4-DUC2 allele (Fig 4). The tract was still called, although ~3*x* shorter (4.6 Kb), at a confidence level greater or equal to 50%. At the lower confidence level, the tract spans over exons 3, 4, 5 and 6 of *RHD* and includes 4 of the 5 key SNPs. The tract was called in 5 out 896 scanned haplotypes: 3 of them were the Papuan carriers of the derived allele at rs150073306 and the 2 remaining ones originated from the Brahui population. All 5 haplotypes carried the derived allele (G) at rs1053355.

## References

1. Bergström A, McCarthy SA, Hui R, Almarri MA, Ayub Q, Danecek P, et al. Insights into human genetic variation and population history from 929 diverse genomes. Science. 2020;367(6484).

2. Delaneau O, Zagury JF, Robinson MR, Marchini JL, Dermitzakis ET. Accurate, scalable and integrative haplotype estimation. Nat Commun. 2019;10(1):5436.

3. Racimo F, Gokhman D, Fumagalli M, Ko A, Hansen T, Moltke I, et al. Archaic Adaptive Introgression in TBX15/WARS2. Mol Biol Evol. 2017;34(3):509-24.

4. Prüfer K, Racimo F, Patterson N, Jay F, Sankararaman S, Sawyer S, et al. The complete genome sequence of a Neanderthal from the Altai Mountains. Nature. 2014;505(7481):43-9.

5. Wagner FF, Flegel WA. The Rhesus Site. Transfus Med Hemother. 2014;41(5):357-63.
