# Supplementary material for: Blood groups of Neandertals and Denisova decrypted
Source: PLoS One. 2021 Jul 28;16(7):e0254175. doi: 10.1371/journal.pone.0254175 (PMC8318287; doi:10.1371/journal.pone.0254175)
Supplement: S1 Table — (DOCX) [file pone.0254175.s004.docx]

S1 Table: Information about the individual analysed in this study.

| **Name** | **Species** | **Localization** | **Age range (kya)** | **Sex** | **Genome coverage** | **Reference** | **Genomic data location** |
| --- | --- | --- | --- | --- | --- | --- | --- |
| "Pinky" Denisova 3 | Denisovan | Denisova cave, Siberian Altaï mountain | 70-90 | Female | 30 X | [12] | <http://cdna.eva.mpg.de/denisova/>  <http://ftp.eva.mpg.de/neandertal/Vindija/VCF/indels/> |
| Altai | Neanderthal | Denisova cave, Siberian Altaï mountain | 120-130 | Female | 52 X | [13] | <http://cdna.eva.mpg.de/neandertal/altai/AltaiNeandertal/>  <http://ftp.eva.mpg.de/neandertal/Vindija/VCF/indels/> |
| Vindija 33.19 | Neanderthal | Croatia | 50-65 | Female | 30 X | [14] | <http://cdna.eva.mpg.de/neandertal/Vindija/>  <http://ftp.eva.mpg.de/neandertal/Vindija/VCF/indels/> |
| Chagyrskaya 8 | Neanderthal | Chagyrskaya cave, Siberian Altaï mountain | 80-90 | Female | 28 X | [15] | <http://cdna.eva.mpg.de/neandertal/Chagyrskaya/> |
